# BioRel: towards large-scale biomedical relation extraction

**DOI:** 10.1186/s12859-020-03889-5

**Published:** 2020-12-16

**Authors:** Rui Xing, Jie Luo, Tengwei Song

**Affiliations:** State Key Laboratory of Software Development Environment, School of Computer Science and Engineering, Beihang University, No. 37 Xueyuan Road, Haidian District, Beijing, 100191 China

**Keywords:** Distant supervision, Relation extraction, Information extraction, Medline

## Abstract

**Background:**

Although biomedical publications and literature are growing rapidly, there still lacks structured knowledge that can be easily processed by computer programs. In order to extract such knowledge from plain text and transform them into structural form, the relation extraction problem becomes an important issue. Datasets play a critical role in the development of relation extraction methods. However, existing relation extraction datasets in biomedical domain are mainly human-annotated, whose scales are usually limited due to their labor-intensive and time-consuming nature.

**Results:**

We construct BioRel, a large-scale dataset for biomedical relation extraction problem, by using Unified Medical Language System as knowledge base and Medline as corpus. We first identify mentions of entities in sentences of Medline and link them to Unified Medical Language System with Metamap. Then, we assign each sentence a relation label by using distant supervision. Finally, we adapt the state-of-the-art deep learning and statistical machine learning methods as baseline models and conduct comprehensive experiments on the BioRel dataset.

**Conclusions:**

Based on the extensive experimental results, we have shown that BioRel is a suitable large-scale datasets for biomedical relation extraction, which provides both reasonable baseline performance and many remaining challenges for both deep learning and statistical methods.

## Backgrounds

In recent years, we have witnessed a rapid growth in biomedical literature. Cohen and Hunter [1] provide an explanation on why the growth in PubMed and Medline publications is phenomenal. However, all the biomedical knowledge in these publications is expressed in the form of unstructured text, which cannot be easily utilized by computer programs. In fact, it is also very hard to manually transform all these knowledge in publications into structured form due to the large quantity of publications. Hence, automated text processing methods for transforming knowledge in text form into machine understandable format are getting more and more attention in biomedical science these days.

Relation extraction, a fundamental technique in Natural Language Processing (NLP), is very suitable to fulfill this task. It would be much easier for biomedical scientists to read and adjust new publications quickly by providing relations between certain entities, for example, possible treatment relation between **ciprofloxacin** and **pyelonephritis**. Plenty of recent studies in relation extraction adopted distant supervised paradigm [2]. Distant supervision aligns entity mentions in plain texts with those in knowledge bases to automatically generate relation instances. It assumes that if two entities have a certain relation in knowledge bases, then all sentences containing this entity pair will express that relation.

Previous studies adopted conventional statistical and graphical methods [3, 4] to detect relations. However, the main disadvantage is obvious in such pipeline since these traditional features explicitly derived from NLP tools can cause error propagation. As deep learning techniques [5, 6] have been widely studied and adopted, plenty of work applied deep neural network for distant supervised relation extraction. Zeng [7] proposed piecewise convolution neural network to build sentence representations and incorporated it into Multi-Instance Learning framework. Lin [8] expected to dynamically reduce the weights of those noisy instances and proposed selective attention over instances. Ji [9] developed a similar attention strategy together with entity descriptions to calculate weights over sentences. Liu [10] proposed a soft-label method to reduce the influence of entity-level noisy instances. Jat [11] used entity-based attention on word-level for efficient relation extraction. Since self-attention mechanism is proved to be effective and efficient, Du [12] started to use structured word-level self-attention and sentence-level attention mechanism to learn rich aspects of sentence representations.

Various relation extraction datasets have also been created in recent years. Doddington [13] introduced ACE 2004, Walker [14] built ACE 2005 dataset for relation classification, and Hendrick [15] proposed SemEval-2010 Task 8. All these datasets aimed at extracting relations in general domain such as news and web. In biomedical relation extraction, there are some widely used dataset such as Bacteria Biotope subtask (BB3), Seed Development subtask (SeeDev), Genia Event subtask (GE4) which is proposed in BioNLP 2016 Shared Task, BioNLP 2019 Shared Task,^1^ Drug–Drug Interaction (DDI) and Chemical Disease Relation (CDR). However, the main drawback is that these datasets are still manually labeled, requiring too much effort from linguists and biomedical experts, which also limits their scales. Besides, there are still some other problems which worth noticing in biomedical relation extraction. (1) Although these statistical approaches achieve promising results, they are still far from satisfactory. (2) Moreover, with the development of deep learning, the scale of human-annotated data is not large enough for deep learning model training and evaluation. (3) The total amount of training and testing instances is also limited in these datasets, which cannot reflect some relation aspects mentioned in the biomedical publications. All these raise the need for creating a large-scale and high quality biomedical dataset for distant supervised relation extraction.

Therefore in this extended version of our previous conference paper [16], we propose the Biomedical Relation Extraction Dataset (BioRel), a large-scale dataset for biomedical relation extraction. BioRel is constructed through distant supervision process, adopting Unified Medical Language System (UMLS) [17] as Knowledge Base (KB) and Medline as corpora resources. UMLS contains information of large amount of various biomedical entities and a wide range of relations. It is not only a rich knowledge base, but also a powerful tool for biomedical text processing. Medline is the U.S. National Library of Medicine (NLM) premier bibliographic database which contains more than 25 million references to journal articles in life sciences with a concentration on biomedicine. Medline is freely available on the Internet. Some selected instances and their entity and relation labels are shown in Table 1.

**Table 1:** Some examples in BioRel dataset

Relation	Sentence
Anatomic structure has location	A brain mass and a **spinal cord** were identified in the **cranial cavity** and the vertebral canal
Therapeutic class of	The histamine induced facilitation was blocked completely by cimetidine and **antidepressant drugs** imipramine and **desipramine**, but remained unaffected in mice pretreated with mepyramine or atropine
Has physical part of anatomic structure	In **normal fibroblastoid cells** 30 min after cultivation the cortical layer would be well defined and demarcated from the adjacent cytoplasma, microfibrillae constituting it are parallel to one another and perpendicular to the cell **membrane**
May treat	Treatment with oral **ciprofloxacin** should offer substantial cost savings over a variety of parenteral antimicrobial regimens (e.g. aminoglycoside + beta-lactams) for difficult to treat infections such as chronic **pyelonephritis**, osteomyelitis, and skin structure infections
May be treated by	The ventricular effective refractory period, as well as the **vt cycle length**, increased after **propranolol** and was further prolonged after the addition of a type i agent

In order to assess the quality of our BioRel dataset, a variety of conventional statistical models and deep learning approaches are re-implemented and deployed as baselines for distant supervised relation extraction. Experiments on BioRel show that deep neural network (DNN) based approaches have better performance in comparison to statistical approaches, which demonstrates the capability of BioRel as a dataset for training deep neural network models. The differences of performances between models which are built to alleviate mislabeling problems are marginal, indicating that the mislabeling problem in BioRel is less serious in comparison to that in datasets of general domain such as NYT.

To summarize, the contributions of our work are as follows:

- We proposed a large-scale dataset for distant supervised biomedical relation extraction;
- We successfully adapt many state-of-the-art statistical and deep learning based approaches to BioRel;
- We conduct comprehensive evaluation of various baselines on our dataset, which indicates that BioRel has less noisy instances and is suitable for both deep learning and statistical based methods by providing reasonable baseline performance and many remaining challenges.

## Results

Our experiments are intended to demonstrate and evaluate the quality of our dataset. In this section, comprehensive experiments are conducted to this end. We first introduce distant supervised relation extraction task formulation. Next, we describe overall experimental settings including word embeddings and parameter settings. And then we compare performance of several state-of-the-art methods on BioRel.

### Task formulation

Multi-Instance-Learning (MIL) is a widely-used framework adopted by most state-of-the-art models in distant supervision. In MIL, the training set are separated into *n* bags $\left\{ \langle {h_1, t_1}\rangle , \langle {h_2, t_2}\rangle , \ldots , \langle {h_n, t_n}\rangle \right\}$, each of which contains *m* sentences $\left\{ s_1, s_2, \ldots , s_m\right\}$ mentioning same head entity $h_i$ and tail entity $t_i$. Each sentence consists of a sequence of *k* words $\left\{ x_1,x_2,\ldots ,x_k\right\}$. For example, the following two instances containing the same entity pair in a bag, are sharing the same relation **may be treated by**. Instance 1: “Three cases of **acute myeloid leukaemia** developing after treatment of renal disease with **cyclophosphamide** have been studied .” Instance 2: “Previous clinical trials have reported that **cyclophosphamide** can be used for the treatment of **acute myeloid leukaemia**.” It’s worth noting that the number of sentences *m* is not always the same in each bag.

### Experimental settings

For most the state-of-the-art neural network models achieving promising results, a cascade of commonly used approaches are adopted, which proved to be effective among various works [8, 9, 11]. To begin with, sentence representation $s_i$ is acquired using encoders on words $\left\{ x_1,x_2,\ldots ,x_k\right\}$, each of which is a word embedding. Then, bag representation $b_i=\langle {h_i,t_i}\rangle$ is produced using sentence representations within. For example, selective attention mechanism is first adopted in [8]. Next, fully connected network is trained as classifier for relation classification on bag level.

**Word Embeddings** Most previous work adopted word embeddings which are traditionally computed from a large corpus of unlabeled text, ignoring domain specific knowledge within. This information can potentially greatly improve the quality of word representation. Therefore in this work, we adopt BioWordVec [18] as word embeddings for all neural model baselines. BioWordVec is an open set of biomedical word embeddings that combines subword information from unlabeled biomedical text with a widely used biomedical controlled vocabulary called Medical Subject Headings (MeSH). The advantage of using BioWordVec is that it contains information in biomedical domain specific structured resources.

Position embeddings are initialized with Xavier for all baseline models. Word embeddings of blank words are initialized with zero while unknown words are initialized with the normal distribution of which the standard deviation is 0.05.

**Table 2:** Parameter settings

Settings	CNN	PCNN	GRU	MIML	MultiR	Mintz
Batch size	256	128	128	–	–	–
Epoch	20	20	20	10	15	15
Learning rate	0.4	0.2	0.3	–	–	–
Word dim	200	200	200	–	–	–
Position dim	10	10	10	–	–	–
Sentence dim	230	230	230	–	–	–
Window size	3	5	–	–	–	–
Dropout	0.5	0.5	0.3	–	–	–

**Parameter settings** Cross-validation strategy is adopted to determine the parameters for our baseline models. And grid search is used to select learning rate $\lambda$ among $\{0.2, 0.3, 0.5\}$, sliding windows size among $\{3, 5, 7\}$, sentence embedding size among $\{100, 200, 300, 400\}$ and batch size among $\{32, 64, 128, 256\}$. Other parameters proved to have little effect on results. Our optimal parameter settings are shown in Table 2.

**Fig. 1:**
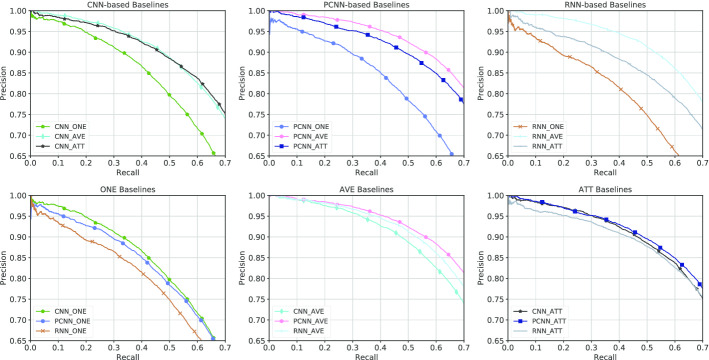
Precision/recall curves of CNN, PCNN and GRU-based models

### Baseline models

**Mintz** Mintz [2] first proposed distant supervision and considered it as a multi-class classification problem. In this work, standard lexical as well as syntactic features are utilized. And for each entity pair that appears in Freebase, all sentences containing this entity pair are collected and then used to train a relation classifier. Lexical features describe specific words between or surrounding an entity pair in the sentence, including the sequence of words between two entities, the part-of-speech tags of these words, a window of *k* words to the head entity and another window of *k* words to the tail entity.

**MultiR** Hoffmann proposed MultiR [19], a probabilistic, graphical model which takes positive and negative bags as input rather than single sentences. MultiR adpoted Multi-Instance-Learning framework, which means that a bag will be labeled positive if it contains as least one positive example, otherwise it will be labeled negative if it contains no positive instance at all. MultiR is freely available and also adapted to our BioRel dataset in this paper.

**MIMLRE** Surdeanu [4] proposed a novel approach for multi-instance multi-label relation extraction. This approach built models for all instances sharing the same entity pair in the text, and also utilized a graphical model with latent variables to classify their relations. Instead of using original data published [4], we adapted the model to our BioRel dataset.

**CNN** Zeng [20] applied deep neural network to this task, which adopted Convolution Neural Networks (CNN) as encoders to extract sentence level features and used pre-trained word embeddings on large unlabeled corpus. This work proposed Positional Embeddings which proved to be effective. Positional Embeddings is defined as the combination of relative word distances between the current word to head entity $e_1$ and tail entity $e_2$. Zeng [20] randomly initializes position embedding matrices and then encodes these distances into vectors. This model is re implemented under MIL framework to fit our own task on BioRel.

**PCNN** Zeng [7] proposed Piecewise Convolution Neural Networks (PCNN). This work treated distant supervised relation extraction as a multi-instance problem and also adopt CNN as sentence encoders. A great contribution of this work is piecewise max pooling technique. This method divides each sentence into three segments based on entity positions, enabling the model to capture structural information for relation extraction. Zeng also demonstrated that incorporating multi-instance learning can effectively solving the wrong label problem and further improve the performance.

**PCNN+Selective Attention** Lin [8] proposed a sentence-level attention-based approach for distant supervised relation extraction. The model embedded the sentence with CNN and built selective attention mechanism over multiple instances. Attention mechanism (ATT) dynamically adjusts weight $\alpha$ for each sentence. In this method, each sentence is first encoded into a vector representation, afterwards, the bag representation is calculated by taking an attention-weighted average of all the sentences in that bag. Comprehensive experiments under “only-one most likely sentence” (ONE) and “average vector over all instances” (AVE) environments are also conducted. In our work, we also follow these experiment environment settings (AVE, ATT, ONE) for better comparison.

**Table 3:** P@N for distant supervised relation extraction models on BioRel

Model	P@4000 (%)	P@8000 (%)	P@12000 (%)	P@16000 (%)	Mean (%)	F1	AUC
CNN+ONE	93.38	84.91	75.00	65.69	79.75	0.66	0.70
CNN+AVE	94.00	90.95	81.58	71.97	85.30	0.72	0.79
CNN+ATT	96.40	90.59	82.35	72.31	85.41	0.72	0.78
PCNN+ONE	92.15	83.80	74.53	65.46	78.98	0.65	0.69
PCNN+AVE	96.57	93.60	85.74	75.39	88.12	0.76	0.82
PCNN+ATT	96.15	91.11	83.27	73.40	85.98	0.73	0.79
RNN+ONE	88.89	81.05	71.58	63.13	76.16	0.63	0.66
RNN+AVE	96.67	92.83	83.97	73.53	87.00	0.74	0.80
RNN+ATT	94.60	89.63	81.81	72.54	84.65	0.72	0.78
Mintz	79.79	67.08	56.93	49.23	63.25	0.49	0.45
MultiR	72.70	66.93	40.32	20.21	50.04	0.30	0.23
MIML	73.35	59.01	48.63	31.40	53.09	0.43	0.39

**RNN** A large variety of work have been utilizing RNN-based models like LSTM [21] and GRU [22] for distant supervised relation extraction task [9, 11, 12, 23–25]. These are more capable of capturing long-distance semantic features compared to CNN-based models. In this work, GRU is adopted as a baseline model, because it is simpler and faster for model comparison on our large-scale BioRel dataset. Selective attention mechanism is also re-implemented and incorporated into all of previous neural-based baseline encoders. Additionally, “only-one” and “average” settings are also considered to examine the performance of the model and noise in BioRel.

**Fig. 2:**
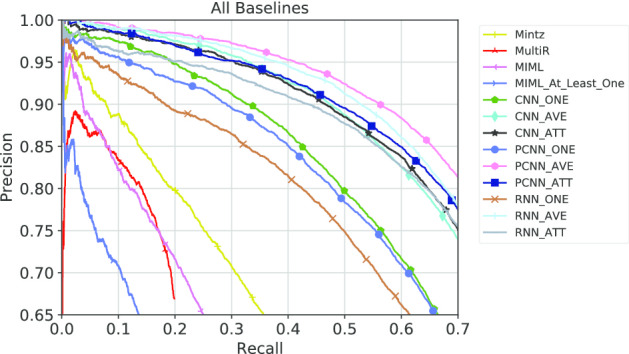
Precision/recall curves of all baselines

### Performance and analysis

Results of all neural network methods are shown in Fig. 1. Our best neural network baselines among “ONE”,“AVE” and “ATT” settings with statistical baselines are shown in Fig. 2. We also report the top precision results, F1-score and AUC of all baseline models in Table 3.

We have the following observations from above : (1) All methods achieve low performance under “ONE” setting, which demonstrates that only selecting “one most likely” sentence is less likely to obtain enough information, and failed to further obtain its bag representation. (2) Even when the recall is over 0.6, all neural baselines have reasonable precision over the entire range of recall, indicating that deep neural networks are effective in detecting various medical relations in our BioRel dataset. (3) Selective attention mechanism proved to be effective in general domain [8], and we expect models to perform better under “ATT” settings than under “AVE” settings. However, as can be seen from the Table 3, CNN and PCNN models have similar performance under “AVE“ and “ATT” settings and GRU model performs even better under “AVE” than “ATT” settings. This indicates that attention mechanism for noise reduction tends to learn an average distribution of sentence representations within bags, which further proves that BioRel maintain high quality relation facts. (4) When recall is smaller than 0.05, all models have reasonable precision among all baselines. While recall is higher, precision of feature-based models decrease sharply compared to neural network-based methods, the latter outperforms the former over the entire range of recall. It demonstrates that human-designed features are limited and cannot concisely express semantic meaning of sentences.

## Discussion

In this section, we analyze BioRel from different angles for a deeper understanding of our dataset.

### Data size

Statistics of BioRel and some widely-used typical Relation Extraction datasets are shown in Table 3. These datasets include SemEval-2010 Task 8, ACE 2003-2004, NYT, BC5CDR, BB3, SeeDev, GE4, i2b2 2010. The first three datasets are used for general-purpose relation extraction and the remaining for biomedical domain. From the table we find that BioRel is larger than existing datasets in both the total amount of words, entities and relations. We expect the large-scale BioRel dataset could facilitate deep learning methods in biomedical relation extraction.

Table 4 shows statistics of BioRel and some widely used representative relation extraction datasets.

**Table 4:** Statistics of relation extraction datasets

Dataset	Word	Sentence	Entity	Relation
SemEval-2010	205k	10,717	21,434	9
ACE 2003-2004	297k	12,783	46,108	24
NYT	21,457k	695,059	17,816	54
BC5CDR	282k	11,089	29,271	1
BB3	34k	1394	2903	1
SeeDev	43k	1549	7082	22
GE4	134k	5130	13,012	5
i2b2 2010	91k	6310	8296	11
BioRel	26,166k	533,560	69,513	125

### Named entity types

BioRel contains a variety of entity types, including clinical drugs, pharmacologic substance, organic chemical, disease or syndrome, biologically active substance, molecular function, food, organ or tissue function and neoplastic process. We linked entities to their Concept Unique Identifiers (CUI) in UMLS and maintain their original expressions in sentences to provide challenges for models.

### Relation types

BioRel consists of 124 labels corresponding to actual relations and a NA (Not A relation) label that indicates there is no relation between two entities. BioRel covers a wide range of relations, involving treatment, component of, side effect, metabolic mechanism, print names, etc. In particular, sentences with NA label account for nearly half of the dataset, which reflects the sparseness between entities of interest in real scenarios, i.e. assuming we randomly select two entities from certain biomedical publications, they are more likely to have no relation of interest. Therefore, we keep a large number of NA instances not only for making high quality model training, but also for making dataset realistic. Moreover, we take a certain relation attributes such as symmetric and asymmetric into consideration to provide further challenges for models. For example, we have both “may treat” and “may be treated by” relations in BioRel, as shown in Table 1.

### Sentence Instances

The training set contains 534,406 sentences and was divided into 39,969 bags, validation set contains 218,669 sentences and 15,892 bags and testing set contains 114,515 sentences and 20,759 bags. Each bag contains sentences sharing the same head and tail entities. The average number of sentences in each bag is 13 in training set, 5 in validation set and 8 in testing set.

## Conclusions

In this paper, we proposed a new large-scale dataset, BioRel, for distant supervised biomedical relation extraction. The dataset was created by aligning UMLS relations to Metamapped Medline Corpus. Relations which may cause false positive are filtered. Meanwhile, we manually kept strict order of relation between medical entities to provide challenges for models to detect exact relations. Comprehensive experiments conducted on the dataset show that the BioRel dataset is especially suitable for distant supervised biomedical relation extraction with the state-of-the-art neural network-based baseline models. Further analysis also suggested that BioRel may have fewer noisy sentences than the widely-used general-purpose NYT dataset.

BioRel enables several promising research directions in the future. It puts forward challenges to relation extraction models for further improving their performance. UMLS contains a large number of vocabularies, each of which focuses on a specific sub-domain. We manually select NDFRT and NCI for relation extraction to facilitate automatic diagnosis. However, the process of BioRel construction can be replicated on other sub-domains as well. Therefore, other vocabularies like MeSH can be chosen freely to construct datasets that meet specific requirements of other researches.

Human annotation and evaluation are still essential. More accurate and precise results and analysis could be provided with the help of researchers and medical experts.

## Methods

In this section, we describe the process of creating the dataset in detail. The whole procedure can be divided into the following three steps: data collection, entity recognition and linking, and distant supervision. In the data collection section, Medline and UMLS are used as the data source to provide corpora, entities and relations. Then, in entity recognition and linking part, MetaMap^2^ is adopted to recognize entities in sentences and link them to their unique identifiers. Finally, we obtain our datasets using distant supervision method which automatically aligns relations to Medline corpora.

**Fig. 3:**
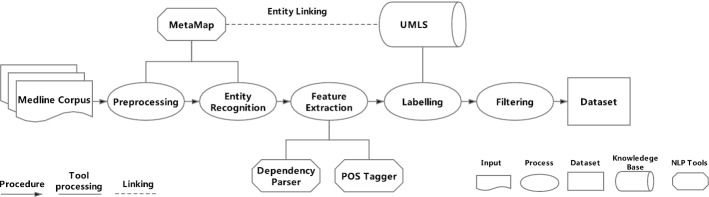
Distant supervision process for BioRel dataset creation

### Data collection

For the first step, we use Medline as the corpus and UMLS as our knowledge base. The data is made freely available on the Internet by the United States National Library of Medicine and can be accessed and searched by the search engine PubMed.^3^ Currently, Medline contains references to more than 25 million journal articles in life sciences with a concentration on biomedicine. In this work, sentences extracted from Medline documents are used to create data and perform extra experiments.

The Unified Medical Language System is a large biomedical knowledge base named Metathesaurus, which contains millions of biomedical entities and relations. MRREL is a subset of the Metathesaurus, which involves only binary relations between different biomedical entities. In this work, we only select two vocabularies as sources: NDFRT (National Drug File - Reference Terminology) and NCI (National Cancer Institute). NDFRT defines the relationship between drugs and diseases, while NCI contains information related to genes and cancer. We choose such selection for two reasons: (a) The number of instances for different relations is unevenly distributed in MRREL. Some relations contain thousands of instances while others only involve hundreds or fewer instances. The knowledge in Metathesaurus is organized into vocabularies related to certain topics. (b) These two vocabularies not only contain enough entities and relations, but also may further assist the development of automatic diagnosis and other technologies by connecting genes, cancer and treatment methods.

### Entity recognition and linking

In order to identify entities in Medline corpus, we adopt MetaMap, a highly configurable program to discover UMLS Metathesaurus entities that referred to biomedical texts. MetaMap uses a knowledge-instensive method based on symbolic, natural-language processing and computational-linguistic techniques. The benefits of using MetaMap are three folds: First, MetaMap is freely available. Second, it has been widely adopted by many works. Finally, it can perform entity recognition and linking simultaneously.

**Table 5:** features for traditional statistical baselines

Lexical	The sequence of words between the two entities
	The part-of-speech of words between the two entities
	A flag indicating which entity came first in the sentence
	A window of k words to the left of the first entity and their part-of-speech tags
	A window of k words to the right of the second entity and their part-of-speech tags
Syntactic	A dependency path between the two entities
	Part-of-speech of words in dependency path
	A ‘window’ node that is not part of the dependency path

### Feature extraction

As shown in Table 5, in order to provide designed features for statistical relation extraction baselines, we extract lexical and syntactic features described in [2, 4, 19]. To take advantage of recent achievement, instead of using traditional statistical NLP tools, we adopt the deep neural network based StanfordNLP [26] tool for part-of-speech (POS) tagging and dependency parsing.

### Distant supervised annotation and filtering methods

To introduce distant supervision in detail, we first traverse the entire Medline corpus and use Metamap to identify medical entities in sentences. Then, we enumerate all combinations of entity pairs in the sentence to create a candidate set. Similarly, relation triples in Metathesaurus are selected to form a knowledge base relation set. Finally, we remove some invalid instances that would affect the performance.

Distant Supervised annotation labels the relations between aligned entities in sentences according to the knowledge base. It assumes that if two entities have a relation in the knowledge base, all sentences containing the same two entities will express that relation. Formally, for each sentence in Medline containing head and tail entities $e_1$ and $e_2$, if there exists a relation triple $(e_1, e_2, r)$ in Metathesaurus indicating that $e_1$ and $e_2$ have the relation *r*, we use the label *r* to denote the sentence containing this entity pair. For example, **ciprofloxacin** and **pyelonephritis** has the relation **may treat** in the knowledge base, then the sentence “Treatment with oral **ciprofloxacin** should offer substantial cost savings over a variety of parenteral antimicrobial regimens for difficult to treat infections such as chronic **pyelonephritis**, osteomyelitis, and skin structure infections.” and “Ciprofloxacin has some effect on pyelonephritis.” would both be labeled as **may treat** relation.

Although efficient and effective, distantly labeled data often contains false positive and false negative instances. We apply further filtering methods to remove some mislabeling data. First, sentences containing entity pairs with two mentions of the same entities are removed. According to Bobic [27], self-relations, i.e. relations between two different aliases of the same entities, are more likely to produce false positives. Second, low-frequency entity pairs are discarded, because entity pairs which occur only a few times within millions of Medline sentences may cause bias in training process. The whole procedure is shown in Fig. 3.

### Evaluation methods

In order to train and test relation extraction models, we divide the training, validation and testing set by randomly selecting instances of each relation from raw dataset. In each relation, 70% of instances are selected as training set, 15% of instances are selected as validation set, and the remaining 15% are used as testing set. Following previous work [2, 8, 10], we evaluate the baseline models on our dataset in the held-out evaluation which provides an approximate measure of precision without requiring expensive human evaluation. We draw precision-recall curves for all models and also report the Precision@N results.

## Data Availability

NYT-10 data used in this paper come from study [3], which can be downloaded from http://iesl.cs.umass.edu/riedel/ecml/. Medline corpora data can be downloaded from https://www.ncbi.nlm.nih.gov/pubmed/ and Metamap can be downloaded from https://metamap.nlm.nih.gov/. The BioRel dataset in this paper is available at https://bit.ly/biorel_dataset.

## Abbreviations

UMLS: Unified Medical Language System
CDR: Chemical Disease Relation
NYT: New York Times
NLM: National Library of Medicine
NDFRT: NationalDrug File - Reference Terminology
NCI: National Cancer Institute
CNN: Convolution Neural Network
PCNN: Piecewise Convolution Neural Network
RNN: Recurrent Neural Network
LSTM: Long Short Term Memory Networks
GRU: Gated Recurrent Units
P@N: Top N Precision
AUC: Area Under the receiver operating characteristic Curve
DDI: Drug–Drug Interaction

## Footnotes

1 https://2019.bionlp-ost.org/.

2 https://metamap.nlm.nih.gov/.

3 https://www.ncbi.nlm.nih.gov/pubmed/.
